# Prehospital emergency nurses’ response: using the socioecological framework to guide health policy recommendations

**DOI:** 10.1186/s13584-025-00708-1

**Published:** 2025-07-16

**Authors:** Rinat Avraham, Yael Wittenbetg, Lior Gal, Odeya Cohen

**Affiliations:** 1 Department of nursing, Recanati School for Community Health Professions, Faculty of Health Sciences, Ben-Gurion University of the Negev, P.O Box 653, Beer Sheva, 8410501 Israel; 2 Emergency Medicine Department, Soroka Medical Center, P.O Box 151, Beer Sheva, 8410101 Israel; 3 Intensive Care Unit, Soroka Medical Center, P.O Box 151, Beer Sheva, 8410101 Israel

**Keywords:** Disaster nursing, Mixed methods, Health policy, Prehospital emergency care, Socioecological framework

## Abstract

**Background:**

Nurses play a vital role in disaster response during emergencies. Nevertheless, limited attention has been paid to factors that influence nurses’ responses and challenges in prehospital settings. These issues became evident during the October 7, 2023, terror attack on Israel, when nurses heroically provided medical treatment, but there was no organized nurse-led initiative to provide emergency care in a prehospital setting.

**Aims:**

(1) To examine the factors associated with nurses’ intentions to provide prehospital emergency reponse during disasters; and (2) To understand multilevel determinantsof nurses’ prehospital emergency response to inform health policy recommendations.

**Methods:**

This study employed an explanatory sequential mixed-methods design. Between February and December 2024, a self-reporting questionnaire was distributed to Israeli nurses (*n* = 315), followed by a qualitative phase involving an open-ended questionnaire completed by 20 healthcare professionals involved in medical care during the attack or in senior emergency preparedness roles. Descriptive and inferential statistics and qualitative content analysis were employed. We applied the socioecological framework to organize the results from both phases.

**Results:**

High personal resilience, readiness and self-efficacy, along with positive attitudes, low hesitancy, and residence in a rural-type settlement significantly predicted nurses’ intention to provide prehospital emergency care. Qualitative analysis revealed four key themes related to nurses’ prehospital roles: (1) individual barriers and facilitators, (2) interprofessional relationships and teamwork, (3) nurses’ roles within the community, and (4) organizational and policy challenges. Findings from both phases were synthesized using the socioecological framework for analysing prehospital nursing care during emergencies.

**Conclusion:**

Nurses’ prehospital emergency response intentions are shaped by personal, professional, and policy-level factors. Beyond education, targeted health policies must clearly define nurses’ roles, strengthen interprofessional collaboration, and integrate nursing into disaster preparedness frameworks to improve system resilience and patient outcomes, particularly in the face of escalating environmental crises globally.

**Supplementary Information:**

The online version contains supplementary material available at 10.1186/s13584-025-00708-1.

## Introduction

One of the most critical components in emergency responses to natural and human-caused disasters is the health system [1]. The role of healthcare systems during emergencies extends beyond providing medical treatment, as sustaining healthcare services is critical for ensuring public safety and enhancing the resilience of affected communities [2, 3]. As they comprise the largest segment of the healthcare workforce, nurses are central to disaster response and recovery efforts [4, 5]. Their strong presence in the community and the trust they command position nurses as key players in disaster preparedness, emergency response, recovery, and mitigation while simultaneously managing chronic diseases (e.g [2]).

Disaster nursing is a specialized field in healthcare, developed in response to the increasing frequency and intensity of disasters in recent decades [6, 7]. Over the years, disaster nursing has shifted from a reactive practice to a proactive field centered on preparedness, integrated policy, and competency-based education. The International Council of Nurses (ICN) laid the groundwork for these efforts by defining competencies spanning mitigation, preparedness, response, and recovery [7–9]. Recent work has situated disaster nursing within broader policy frameworks, advocating for the development of structured guidelines on leadership, ethical protocols, and adaptations to preparedness policies. A historical analysis by Fletcher and colleagues (2022) traced the shift from short-term crisis response to proactive disaster risk management while highlighting gaps in workforce training and advocacy [10]. According to these authors, additional research and increased representation of the profession at a strategic and political level could enhance the effectiveness of nurses’ roles in emergencies.

On October 7th, 2023, Israel suffered its deadliest attack in the past 50 years in terms of fatalities per capita. Approximately 3,000 Hamas-led terrorists infiltrated Israel via the southern border from Gaza, targeting civilians in their homes, an outdoor music festival, and military bases. The terrorists invaded 20 communities and several Israel Defense Forces bases, using assault rifles and explosives, which resulted in hundreds of casualties [11]. At the music festival, attended by approximately 4,000 people, 364 were killed and hundreds more injured [12]. In total, almost 1,200 people were killed by firearms, explosives, decapitation, mutilation, or burning. Additionally, hundreds were abducted, and thousands more were injured. According to Goldman and colleagues (2024), the National Center of Forensic Medicine was tasked with identifying almost 1,200 victims in a single day [11]. On October 7 and 8, 2023, approximately 2,000 injured individuals were treated at emergency departments [12, 13].

According to the Division of Nursing of the Ministry of Health [14], on the first day of the attack, at least 10 local nurses heroically provided medical treatment to numerous casualties, exposing themselves to extreme danger. Another nurse, who was at a military base, treated injured soldiers while sustaining injuries herself. One nurse who was taken hostage in Nir Oz continued to care for her fellow hostages, most of whom were elderly kibbutz members, during a 54-day period of captivity underground in Gaza. However, based on a review of professional experiences and reports from healthcare personnel during this period, no evidence was found of an organized nurse-led initiative that provided emergency care during the prehospital phase.

Despite the pivotal role played by nurses in disaster response, the role of a prehospital emergency nurse is not yet established and varied among countries [e.g., 4, 9]. The ICN [8]– [9] provides a set of core competencies relevant to prehospital settings, including leadership in disaster planning and drills, rapid assessment and triage, effective communication within emergency teams, and ensuring responder and patient safety alongside ethical practice. However, in Israel there is no definitive role for nurses in the prehospital phase.

Furthermore, the factors affecting nurses’ preparedness, challenges, and intention to respond during disasters remain limited [15, 16]. Tas and Cakir (2022) found that nurses are not sufficiently prepared for disasters and lack confidence in responding effectively to disasters [17]. Farokhzadian and colleagues (2024) highlighted nurses’ inadequate skills in responding to disasters [18]. Sani Mert and Koksal (2024) found that nurses’ sense of moral obligation, insufficient experience, balancing of responsibilities, and preparation challenges influenced the difficulties they faced and their motivation in responding to disasters [15]. To address this knowledge gap, the aims of the present study were as follows: First, to examine the factors associated with nurses’ intentions to respond during emergencies in prehospital settings, in terms of their attitudes, knowledge, resilience, and altruism. Second, to understand the multilevel determinants of nurses’ prehospital emergency response for developing health policy recomendations.

## Materials and methods

### Design

An explanatory sequential mixed-methods design was employed, in which we first collected and analyzed quantitative data, followed by the collection and analysis of qualitative data to further clarify and deepen our understanding of the quantitative findings [19]. For the quantitative phase of the study, we employed a cross-sectional design and distributed an anonymous online questionnaire. During the qualitative phase, we used an open-ended online questionnaire distributed to key informants.

### Participants and settings

Quantitative phase: Between February and July 2024, nurses from across Israel were invited to participate in our survey. Participants were recruited using an online, snowball convenience sampling method. Eligibility criteria required participants to have been actively employed as a nurse for at least one year. Nurses who were temporarily not working during the October 7, 2023 attack due to medical or personal reasons were excluded. A total of 315 nurses completed the survey.

Qualitative phase: The open-ended questionnaire was distributed between August and December 2024. To ensure the inclusion of individuals who could make meaningful contributions to the understanding of the research topic, participants were selected based on criteria derived from the researchers’ professional background [19]. RA is a nurse living in a community of the Gaza envelope, and OC is a nurse expert in emergency preparedness and response. We reached these participants following their expertise and experience, publications, and previous information we had about their participation in prehospital care. We targeted two groups of participants:

1. Nurses and paramedics who were actively involved in providing medical care during the October 7th attack.
2. Healthcare professionals, who determine the roles of nurses during emergencies and their emergency preparedness.

### Measures

During the quantitative phase, a self-reporting questionnaire was used to assess participants’ attitudes and intentions regarding their responses to an emergency, resilience, and altruism. In addition, participants were asked to provide any further relevant details, including previous experience and training they had received in emergency response.

#### Perceptions and intentions

Cognitive factors influencing nurses’ intention to participate in prehospital treatment efforts in response to local disasters, based on the theory of planned behavior [20], were measured using a valid and reliable measure [21]. This tool included 19 Likert-scale items, ranging from 1 (strongly disagree) to 5 (completely agree), examining (1) attitudes toward the role of nurses in an emergency (knowledge, two items; readiness, two items, and cognitive attitudes, three items); (2) subjective norms, comprising three items to evaluate the perceptions of participants about how those close to them expect them to respond in an emergency situation; (3) self-efficacy, five items, and hesitancy to respond in an emergency situation, two items; and (4) intention to respond in a future emergency situation, two items.

#### Personal resilience

Personal resilience was measured using the Connor-Davidson Resilience Scale (CD-RISC) questionnaire [22]. Individual resilience refers to an individual’s ability to successfully cope with challenging events and return to their previous functionality in the shortest possible time. In this study, we used a shortened version of the questionnaire, which included ten items that measure a sense of individual resilience in the face of difficulties. The items were rated on a Likert scale ranging from 0 (never true) to 4 (almost always true).

#### Community resilience

Community resilience was measured using the Conjoint Community Resilience Assessment Measure (CCRAM) questionnaire [23]. Community resilience refers to people’s trust in local leadership, collective efficiency, emergency preparedness, attachment to place, and social trust. In this study, we used a shortened version of the questionnaire, which included ten items (out of 21). These items were rated on a Likert scale ranging from 1 (I do not agree at all) to 5 (I strongly agree).

#### Altruism

Altruism was measured using the Self-Report Altruism Scale (9-SRA) [24]. Altruism refers to human behavior characterized by unconditional generosity and placing the needs of others before one’s own. We used a shortened version of the SRA, which included nine items rated on a 5-point scale ranging from 1 (never) to 5 (always). We differentiated between two subscales: (1) altruism—helping others (six items) and (2) altruism—charitable donations (three items), with each subscale calculated as an average score.

#### Personal and professional characteristics

Personal and professional characteristics were also collected, including age, gender, district of residence, type of settlement (urban or rural), level of academic and professional education, seniority in the nursing profession, previous experience in caring for patients during an emergency, and actual treatment performed on October 7.

#### Qualitative survey

For the qualitative phase, the open-ended online survey comprised three questions designed to elicit detailed and reflective responses. Participants were encouraged to share insights based on their personal experiences and professional perspectives. The three questions were as follows.

1. How do you perceive the role of the nursing profession in responding to emergency situations such as accidents, disasters, and terrorism during the occurrence of these events in the field? In your opinion, what are the unique aspects of the nursing profession during such events? Our intention was to gain insights beyond the medical care provided in healthcare institutions such as hospitals.
2. Can you identify any gaps between your perception of the nursing role in emergencies and the current reality?
3. What is required to bridge any gaps and fully realize your vision of nursing in emergency situations, in terms of education and training, workforce preparedness, and systemic support?

### Data collection

Data collection took place between February and December 2024. The questionnaires for both phases were distributed through social media platforms. For the quantitative data collection, the questionnaires were distributed specifically within nurse-related groups on Facebook and WhatsApp. Participants who were interested in participating provided informed consent before completing a questionnaire. For the qualitative phase, we approached relevant participants and obtained their oral consent to participate. Following this, we sent them the open-ended questionnaires, which they completed anonymously online.

### Data analysis

During the quantitative phase, participants’ characteristics and main variables of interest for our study were analyzed to provide descriptive statistics, using frequencies and proportions or means and standard deviations, depending on the type of variable. Comparisons between groups were performed using independent *t*-tests. Pearson’s correlations were used to analyze associations between the main study variables. Multiple linear regression was employed to analyze factors associated with the intention to provide emergency prehospital care. Statistical significance was defined as *p* < 0.05.

During the qualitative phase, two of the authors (RA and OC) analyzed the survey answers. Content analysis was conducted inductively, drawing on text units [19]. First, we looked for similarities and differences among the participants’ statements. Similar statements were classified under the same category according to their compatibility with the research topic. The collected data were then classified according to themes.

The findings from the quantitative stage were compared and interpreted in conjunction with those from the qualitative phase. The authors actively participated in discussions throughout the integration process. We applied the socioecological framework (SEF) [25] to organize the results from both phases, mapping the levels of influence of individual views, interpersonal and organizational characteristics, and systemic aspects in the provision of prehospital emergency care by nurses during emergency situations. The SEF is a theoretical framework used to analyze how different levels of influence—individual, interpersonal, organizational, community, and policy—affect behaviors, decisions, and outcomes. In healthcare research, this framework is particularly valuable for understanding the multifaceted factors that shape the delivery of care (e.g [26]).,.

### Ethical considerations

Approval for this study was obtained from the Institutional Review Board of Ben-Gurion University of the Negev, approval number #697-2. In both phases, participants were informed of the study’s purpose and provided informed consent to participate. Participation was voluntary, all responses were confidential, and participants could withdraw at any time, for any reason, without penalty.

## Results

### Quantitative phase

#### Characteristics of the quantitative study population

The majority of respondents were women (91%, *n* = 287 out of 315 in total). Approximately 45% of participants were from the southern district of Israel (*n* = 141), most of whom held a baccalaureate degree (59%, *n* = 184) and had advanced training in emergency medicine (58%, *n* = 182). Approximately half of the participants were employed as hospital nurses (*n* = 162), while 20% worked as primary care nurses in the community (*n* = 63). Although more than half of the nurses had received training in emergency response, just one-third played an active role in emergency situations, and just one-quarter responded to the emergency on October 7, 2023. Full sociodemographic details and professional characteristics of the participants are presented in Supporting Table A1.

#### Main study variables

Attitudes (mean = 4.26, SD = 0.733) and intentions (mean = 4.25, SD = 0.671) were rated high, while subjective norms (mean = 3.80, SD = 0.775) and self-efficacy (mean = 3.67, SD = 0.630) were rated lower. Participants reported a medium level of readiness (mean = 3.28, SD = 0.919) and knowledge (mean = 3.69, SD = 0.823), a low level of hesitancy (mean = 2.37, SD = 0.784), and a medium level of personal resilience (mean = 3.75, SD = 0.593), which was higher than their community resilience (mean = 3.38, SD = 0.788). Their level of altruism—charity was higher than their level of altruism—helping others (mean = 3.43, SD = 0.802 and mean = 2.91, SD = 0.744, respectively).

#### Comparisons of the main variables between subgroups

Three comparisons were made between subgroups: those who responded vs. those who did not respond, those who hesitated vs. those who did not hesitate, and those who received training receivers vs. those who did not receive training.

Nurses who responded during the terror attack on October 7, 2023 (*n* = 78) reported a significantly higher level of altruism—helping others (*t* = -2.06, *p* < 0.05) and had a significantly higher level of knowledge (*t* = -2.62, *p* < 0.01), readiness (*t* = -2.66, *p* < 0.01), and personal resilience (*t* = -3.30, *p* < 0.01) compared to nurses who did not respond (*n* = 223). The full comparisons are presented in Supporting Table A2.

Nurses who are hesitant to respond to a disaster (hesitation level > 3.0; *n* = 96) had lower scores in relation to knowledge, readiness, and personal resilience, as well as less positive attitudes, norms, self-efficacy, and intentions (*p* < 0.01), compared to non-hesitant nurses (hesitation level ≤ 3.0, *n* = 219). The differences between groups in terms of community resilience and altruism levels were not significant (*p* > 0.05). A full comparisons are presented in Supporting Table A3.

A comparison between nurses who had received training in emergency care and those who had not received such training (*n* = 182 and 132, respectively) revealed that trained nurses had significantly greater knowledge and readiness (*p* < 0.001), self-efficacy (*p* < 0.01), and intention to respond to a disaster (*p* = 0.018). The full comparisons are presented in Supporting Table A4.

#### Factors related to prehospital emergency response intention of nurses

We also aimed to identify factors associated with nurses’ intentions to respond during prehospital emergency events. Table 1 shows the univariate Pearson’s correlations between the dependent variable (intention to respond) and the independent variables. All of the correlations were significantly associated with the intention to respond (*p* < 0.01).

Table 2 shows our multiple linear regression analysis of nurses’ intention to respond in prehospital emergency event. Sense of readiness and personal resilience, positive attitudes and self-efficacy, and low levels of hesitancy were significant predictors of the dependent variable. In addition, nurses who lived in rural rather than urban settlements showed higher levels of intent to respond. The model accounted for 60.8% of the variance in the nurses’ intentions (F = 26.393, *p* < 0.01).

**Table 1:** Means, standard deviations, and correlation matrix of the main study variables

Variable	Mean (SD)	1	2	3	4	5	6	7	8	9	10
1. Intention	4.25 (0.67)	—									
2. Knowledge	3.69 (0.82)	0.48^**^	—								
3. Readiness	3.28 (0.92)	0.44^**^	0.76^**^	—							
4. Attitudes	4.26 (0.73)	0.60^**^	0.32^**^	0.28^**^	—						
5. Norms	3.80 (0.77)	0.57^**^	0.33^**^	0.34^**^	0.54^**^	—					
6. Self-efficacy	3.77 (0.63)	0.59^**^	0.52^**^	0.49^**^	0.50^**^	0.51^**^	—				
7. Hesitance	2.37 (0.78)	− 0.41^**^	− 0.40^**^	− 0.36^**^	− 0.30^**^	− 0.27^**^	− 0.32^**^	—			
8. Personal resilience	3.75 (0.59)	0.46^**^	0.39^**^	0.37^**^	0.42^**^	0.29^**^	0.46^**^	− 0.31^**^	—		
9. Community resilience	3.38 (0.79)	0.15^*^	0.13^^^	0.14^^^	0.20^**^	0.20^**^	0.11	− 0.11	0.19^**^	—	
10. Altruism– charity	3.43 (0.80)	0.19^**^	0.06	0.07	0.25^**^	0.17^*^	0.12^^^	− 0.11	0.36^**^	0.22^**^	—
11. Altruism– helping others	2.91 (0.74)	0.21^**^	0.22^**^	0.27^**^	0.21^**^	0.21^**^	0.20^**^	-14^^^	0.39^**^	0.12^^^	0.48^**^

^**^p < 0.001, ^*^p < 0.01, ^^^*p* < 0.05

**Table 2:** Multiple regression analysis of nurses’ intention to respond in prehospital emergency event

Variable	β	Standard error	Standardized β	t	*p*-value
Age	-0.003	0.002	-0.055	-1.258	0.21
Training received (yes vs. no)	0.018	0.06	0.013	0.3	0.765
Knowledge	0.014	0.06	0.016	0.232	0.817
**Readiness**	0.119	0.052	0.154	2.261	**0.025**
**Attitude**	0.404	0.055	0.412	7.374	**< 0.001**
**Self-efficacy**	0.255	0.064	0.238	3.984	**< 0.001**
**Hesitance**	-0.107	0.042	-0.119	-2.511	**0.013**
Gender (M vs. F)	-0.141	0.107	-0.062	-1.325	0.187
**Settlement type** **(urban vs. rural)**	-0.158	0.067	-0.108	-2.378	**0.018**
**Personal resilience**	0.137	0.063	0.115	2.177	**0.031**
Community resilience	-0.026	0.04	-0.03	-0.648	0.518
Altruism– charity	0.024	0.044	0.028	0.551	0.582
Altruism– helping others	-0.038	0.049	-0.04	-0.785	0.434

### Qualitative phase

#### Characteristics of the qualitative study population

A total of 20 participants completed the online open-ended qualitative questionnaire. The majority of them were female (70.0%), nurses (60.0%), and with ages ranging from 30 to 68. Most of the participants had prior experience in emergency situations (70.0%), and half of them responded on October 7, 2023. More detailed characteristics of the participants, alongside descriptions of their previous experience, can be found in Supporting Table B1.

#### Themes, categories, and meaning units

**Fig. 1:**
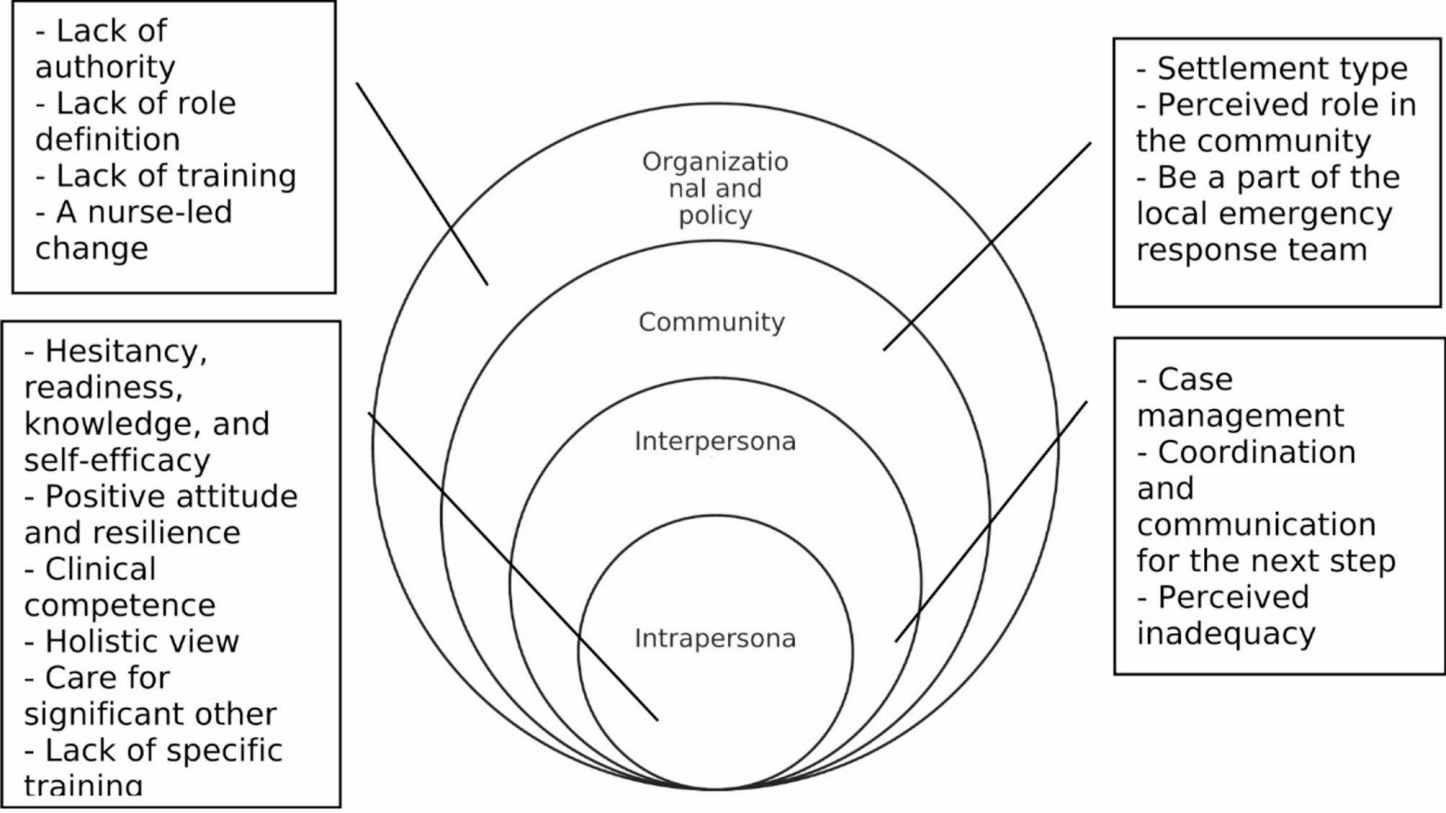
A diagram summarizing the determinants of prehospital emergency care provided by nurses, conceptualized using the socioecological framework

Four main themes emerged from the open-ended questionnaires regarding nurses’ roles in prehospital emergency care: (1) individual barriers and facilitators, (2) interprofessional relationships and teamwork, (3) the nurses’ roles in their communities, and (4) organizational and systemic/policy challenges. We organized the themes and categories according to the SEF, as illustrated in Fig. 1. No substantial differences between nurses and other healthcare professionals were observed.

*Theme 1: Individual barriers and facilitators*.

Participants stated that nursing staff are clinically competent in providing care in emergency situations and therefore must be part of the response team and play a formal role in responding to disasters:

*The uniqueness of the nursing profession in emergency situations lies in their expertise and knowledge in providing life-saving emergency care*,* as well as in their ability to manage stressful events effectively…They [the nurses] bring extensive medical knowledge*,* enabling them to assist in decision-making processes in complex conditions—such as determining whether intubation is necessary*,* whether fluid resuscitation is crucial*,* and more* (P13, Physician).

However, nurses do not receive sufficient training in prehospital emergency care, which hinders their ability and intention to take part in responding to emergency situations and mass-casualty events.

*I believe that additional and advanced medical knowledge can be beneficial. For example*,* performing a thoracostomy or intubation. Such skills can enhance field treatment*,* even in the absence of other medical personnel with the necessary expertise* (P1, Nurse).

Nurses also described having a holistic point of view, which is essential in managing mega-casualty events, which can help prevent short- and long-term complications and influence patients’ recovery from trauma:

*A nurse could take a holistic view of the event*,* identify psychological distress*,* and help prevent PTSD [post-traumatic stress disorder] with proper care* (P16, Nurse).

This sentiment was echoed by another participant, who also referred to the responsibility of nurses to others affected by the emergency situation, such as family members or friends who are worried about their loved ones in a disaster setting:

*A nurse has a broader role that not only ensures and assists in the physical treatment of the injured but also addresses the wider circles and additional emerging needs* (P4, Nurse).

*Theme 2: Interpersonal relationships and teamwork*.

Participants emphasized how the inherent ability of nurses as case managers can be useful in emergencies.

*They [nurses] can effectively mediate the situation for patients and those around them*,* serving as a liaison between the patient and other caregivers in the field* (P5, Nurse).

Nurses also play a crucial role in organizing and relaying information about the injured to ensure a smooth transition to their next point of evacuation.

*The nurse’s approach to providing an initial response in any event…begins with delivering primary life-saving assistance…and communicating information about the injury and the treatment given to ensure continuity of care* (P9, Nurse).

On the other hand, the prevailing perception among healthcare professionals was that nurses lack the knowledge and skills to provide care in the field, a perception that likely affects their involvement in such events.

*There is still a perception that nurses are less professionally skilled compared to emergency medical personnel or paramedics. As a result*,* in the field*,* there may be a tendency to rely on them less if other medical professionals are present* (P5, Nurse).

*Theme 3: The role of nurses in their community*.

A common perception was that nurses are not part of the active team involved in prehospital emergency events, a notion evident both within and especially outside the hospital setting.

*Currently*,* there is no clear*,* structured perception of the role of nurses in emergencies*,* with the gap being most evident outside the hospital setting* (P19, Paramedic).

One participant emphasized the importance of providing targeted training, in collaboration with local emergency response teams, to define the role of nurses in emergencies that occur in their own communities. This training would ensure that nurses understand their responsibilities and are prepared to respond effectively.*In my opinion*,* there is a lack of training for all nurses to be part of local emergency response teams (such as ‘Tzahi’—a local emergency team) and similar units* (P8, Physician).

*Theme 4: Organizational and policy challenges*.

Nurses lack authority outside of their institutions and cannot provide medical care without a doctor’s orders. This limitation may discourage nurses from acting in emergencies due to concerns about inadequate legal protection.

*In emergency situations*,* there are times when life-saving medication is required. Currently*,* medical orders are needed*,* aside from over-the-counter treatments* (P3, Nurse).

There is no professional discussion or policy supporting nursing role in emergencies, unlike other healthcare professionals who have clearly defined roles and are expected to act in such situations.

*There is no clear definition*,* or at least none that I am aware of*,* for the nursing profession in emergencies—unlike other professions such as doctors and paramedics* (P7, Researcher).

Clarifying the authority of nurses in emergency situations and incorporating this topic into the nursing curriculum from the outset could drive changes to the current situation.

*I believe there is a need to develop such a definition and integrate it into the nursing curriculum at the initial training stage…specialty in disaster nursing should be considered* (P20, Nurse).

To advance this process, internal motivation and drive are crucial, with nurses playing a pivotal role in inspiring and leading it rather than relying solely on external forces.

*[It needs] a push from nurses*,* parallel training to that of paramedics*,* and research that will show a clear contribution of the presence of nurses compared to their absence in emergency situations* (P2, Paramedic).

To enhance the credibility of our findings, we find it important to note that, despite the broad agreement among participants regarding the need to redefine and expand the role of nurses in prehospital emergency care, two participants expressed contrasting views. They (P4, P11) stated that no gap exists today, indicating that the current situation is adequate and does not require change.

### Synthesis of quantitative and qualitative results and practical steps toward change

The results from the qualitative phase provided further insights into the information we derived from the quantitative phase, enabling a more comprehensive understanding of the determinants of prehospital emergency care provided by nurses. The combined results from both phases are summarized in accordance with the SEF in Fig. 1. While Fig. 1 highlights the factors that influence the intentions and practices of nurses in response to disasters, some practical suggestions were also proposed, focusing on operational steps to enhance the role of nurses in emergencies. First, appropriate education and training are needed, including simulation training and practice in dealing with serious emergency situations at least once a year. Second, there is a need to regulate authorization for nurses to provide medical care during emergencies and ensure that they have legal protection. Third, medical professionals from various fields should all undergo the same multidisciplinary training to foster unity and a shared sense of purpose. From a broader perspective, a shift in perception is needed to recognize the nursing workforce as an integral part of the medical personnel potentially available for immediate emergency response. This paradigm shift will help drive the changes necessary to effectively implement this approach.

## Discussion

This study pursued two key goals: to examine the factors influencing nurses’ intentions to respond to emergencies in the prehospital setting, and to develop practical health policy recommendations grounded in a multilevel framework. We found that participants who received appropriate training demonstrated greater knowledge, readiness, self-efficacy, and intention. Additionally, nurses who responded on October 7, 2023, demonstrated greater knowledge, personal resilience, and altruism than nurses who did not respond. Personal resilience, a sense of readiness, a positive attitude, self-efficacy, and lower hesitancy significantly predicted nurses’ intentions to provide prehospital emergency care. Our qualitative analysis identified four key themes related to nurses’ prehospital care roles: (1) individual barriers and facilitators, (2) interprofessional relationships and teamwork, (3) nurses’ roles within the community, and (4) organizational and policy challenges. Findings from both phases were synthesized using the SEF to identify multilevel factors and develop a potential approach to improve prehospital care provided by nurses during emergency situations.

### Policy implications and recomendations in line of the socioecological approach

While the SEF has been successfully used to interpret the complex influences on individuals in public health settings, several studies have also employed this framework to outline healthcare interventions, exploring essential factors in complicated circumstances across a wide range of healthcare areas. Employing the SEF can help shed light on how multilevel factors interact to shape a given care setting, offering a structured basis for designing effective, multilevel interventions tailored to diverse healthcare contexts. For instance, Litchfield and colleagues (2021) explored the factors that could influence safe practice in UK primary care when previous initiatives had failed to deliver the expected improvements [26]. Mace et al. (2024) identified barriers of HCP in behavior change-oriented dementia prevention, and proposed strategies for addressing these barriers within the larger contexts of SEF [27]. Ude and colleagues (2022) suggested that the SEF could be used to explore transitioning to adult care in young people with diabetes [28]. Although various aspects from individual to policy levels have been widely discussed, the SEF has yet to be utilized for analyzing the multilevel influences on disaster nursing or informing health policy recommendations. The following sections will discuss the study’s findings, integrating them with existing literature and exploring pathways for shaping health policies that can advance disaster nursing and its impact on population health.

The use of the SEF in this context enables the translation of individual and organizational insights into actionable health policy. Our findings suggest that targeted training programs, role clarification for nurses in prehospital care should be embedded within a structured national strategy for emergency preparedness.

### Intrapersonal level

Nurses play a vital role in disaster preparedness, including identification, risk assessment, strategic planning, conducting drills, participating in training programs, and identifying areas for improvement [29]. However, despite their potential to have a significant impact during disaster response, they are often underutilized. Several critical factors, including inadequate training and insufficient preparedness hinder the participation of nurses in the disaster response phase. Providing emergency care during a disaster is often accompanied by concerns related to the security situation. Furthermore, many nurses feel unprepared and lack the necessary skills to respond effectively during disasters [18]. We found that nurses who hesitated to participate in disaster care had lower intentions to respond.

Self-efficacy - a key determinant of motivation and behavior, plays a crucial role in shaping nurses’ performance in disaster situations, as it reflects their confidence in successfully carrying out essential tasks during emergencies [20]. Our findings align with previous studies demonstrating the positive impact of targeted training on nurses’ self-efficacy in disaster response scenarios. Choi and Lee demonstrated that disaster preparedness training enhances both self-efficacy and disaster management skills among nurses [30]. International research has consistently identified self-efficacy as a key determinant of healthcare workers’ willingness to act in crisis situations [30, 31]. Our findings support the broader literature as they show a clear association between self-efficacy and nurses’ intention to respond during disasters, consistent with the theory of planned behavior. Therefore, strategies aimed at strengthening nurses’ self-efficacy are essential for improving their willingness to participate in disaster response. Greater self-confidence in their ability to respond effectively can be fostered through a policy of ongoing workplace training and dedicated disaster preparedness education during their formal studies. In a brouder perspective, disaster relief requires a comprehensive and coordinated response from healthcare organizations, government agencies, and support systems by providing adequate training, ensuring safety protocols, offering mental health support, and fostering a fair and supportive work environment to mitigate the adverse effects on nurses [15]. It is important to acknowledge the potential for reverse causality.

### Interprofessional level

Interprofessional relationships and teamwork in primary care can have a major influence on healthcare quality, impacting both clinical and humanistic outcomes. Research has demonstrated the effect of formal interprofessional relationships can have on medical errors, patient satisfaction, patient and caregiver education, and mortality [32, 33]. Nurses possess essential teamwork skills for disaster response, including activating disaster plans, triaging patients, and coordinating evacuations and patient transport to medical facilities [18, 34]. Furthermore, nurses play a vital role in addressing the mental health needs of those affected by disasters by providing psychological first aid to patients, families, and significant others [35]. However, due to the infrequent and often undefined nature of the role of nurses in disaster response [36, 37], their integration into multidisciplinary teams involved in prehospital care remains inadequate. A multidisciplinary task force should be established to develop an operational model that incorporates nurses as part of the prehospital emergency response during disasters.

### Community level

Our findings suggest that nurses in rural areas are more likely to respond to disasters within their communities than their urban counterparts. There are several possible explanations for this finding. Rural nurses play a distinctive role compared with their urban and metropolitan counterparts. In many cases, a nurse may be the only clinician present at a rural healthcare facility, with other medical professionals only accessible on an on-call basis [38]. They often have close relationships with their communities prior to a disaster and good knowledge of the community in which they serve, which enables easier healthcare assessment and management, as well as the ability to advocate on the community’s behalf [39]. Rural nurses also demonstrate a commitment and dedication to continue to provide care to the community in which they serve, often despite their personal hardships [40]. Although community resilience was not a direct predictor of disaster response intention, our findings indicate that nurses in rural areas exhibited higher levels of community resilience than nurses in urban areas. Further research is needed to explore the potential indirect relationship among nurses and their intention to respond and their community resilience levels.

Another community-level aspect reflected in our study pertains to the social norms within the nursing profession regarding nurses’ roles in disaster response. The level of professional advancement available for disaster nursing varies across countries, but the initial stage involves recognizing the necessity of disaster nursing, understanding nurses’ skills and their capacity to operate effectively during a disaster, followed by the need for specialized training, role expansion, and leadership in the field [36]. To drive change in this field, nurses should take an active role, advocate for progress, and assume responsibility for the development of their profession.

### Organizational and policy level

Participants considered several policies and organizational aspects essential to improve nurses’ intentions to respond to emergencies. Previous studies have noted that role ambiguity, coordination deficiencies, and systemic challenges can adversely affect nurses’ ability to participate in an emergency response [29, 41]. Xue and colleagues (2020) conducted a meta-synthesis that revealed a lack of clear systems, processes, protocols, and guidelines that affect nurses’ abilities to mount an efficient response [42]. Azizi and colleagues (2021) highlighted that role ambiguity was one of the role stressors among prehospital nurses during disasters [43]. Prehospital settings are characterized by inadequate coordination, resource shortages, understaffing, and unclear responsibilities within interdisciplinary teams [39].

Beyond the need for coordinated and effective teamwork, the lack of disaster-specific content in nursing curricula and its consequent impact on preparedness has been widely discussed. Veenema et al. (2016) identified insufficient education as one of the barriers to advancing professional disaster nursing [5], a concern also raised in several review studies (e.g [29, 42]).,. Furthermore, Al Thobaity (2024) highlighted the importance of education in overcoming challenges related to nursing disaster preparedness and response [41], while Al Harthi and colleagues (2021) noted the limited research and evidence-based practices to support the effectiveness of integrating nurses in disaster response [29]. A recent study suggested that there is an urgent need to improve disaster literacy within the nursing profession, thereby better preparing nurses for disaster situations. The authors identified nine critical dimensions that constitute the disaster literacy of nurses: physical and mental qualities, general knowledge relating to disaster rescue, professional and technical competence, professional ethics, teamwork, emotional ability, information literacy, leadership, and knowledge transformation [44]. While these reviews have not focused solely on prehospital settings, they underscore that the situation in such settings is notably weaker than in the hospital environment.

### Limitations

This study had several limitations. First, we employed a convenience sampling in the quantitative phase, which may resulted in over or under representation of sub groups and limits the generalizability of the findings. In addition, Although we asked participants in the quantitative phase if they responded on October 7, 2023, we were unable to distinguish between nurses who responded independently and those who responded following a call from their employer. In addition, data collection was conducted post-hoc, which may have influenced participants’ responses and limits the ability to infer causality. Thus, we evaluated the participants’ intentions to respond to future events. Furthermore, It is important to acknowledge the potential for reverse causality as the intention to act may itself reinforce perceived self-efficacy, particularly in the context of a cross-sectional design [45]. Future prospective research is recommended.

Second, we used an open-ended survey questionnaire rather than in-depth interviews to identify perceptions and gaps regarding nurses’ roles in disasters, which could potentially limit the possibility of elaborating, clarifying, or further exploring the meanings behind their statements. However, we employed a systematic approach to data coding involving two independent coders to minimize any personal biases in our analysis. Additionally, the use of a mixed-methods approach allowed us to cross-reference insights and assess the validity of our findings.

## Conclusions

Our findings support concrete health policy actions. Policymakers should prioritize the development of national guidelines that formally define nurses’ roles in prehospital care, integrate disaster preparedness into nursing education, and establish support mechanisms to improve nurses’ readiness and willingness to respond. Applying the SEF allowed us to bridge frontline experiences with system-level solutions, offering a roadmap for meaningful policy reform.

Given the increasing frequency of extreme environmental events, urgent action is needed at the policy level to formally recognize and support the critical role of nurses in prehospital emergency preparedness and response. Policymakers should prioritize the development of national guidelines, formalized role definitions, and comprehensive training frameworks that position nurses as integral members of disaster response teams. Advancing these policies will enhance healthcare system resilience, optimize patient outcomes during emergencies, and strengthen the nursing profession both in Israel and globally.

## Electronic supplementary material

Below is the link to the electronic supplementary material.

Supplementary Material 1

## Data Availability

No datasets were generated or analysed during the current study.

## Declarations

### Supporting information

In supporting characteristics of the quantitative study population, detailed descriptions of sociodemographic and professional characteristics of the participants (Table A1) are provided. In supporting comparisons of the main variables between subgroups, the full comparisons are presented in Tables A2, A3 and A4. In supporting characteristics of the qualitative study population, detailed information about the participants, alongside descriptions of their previous experience, is provided (Table B1).
