# Supplementary material for: Prehospital emergency nurses’ response: using the socioecological framework to guide health policy recommendations
Source: Isr J Health Policy Res. 2025 Jul 16;14:44. doi: 10.1186/s13584-025-00708-1 (PMC12265371; doi:10.1186/s13584-025-00708-1)
Supplement: Supplementary file 1 — Supplementary Material 1 [file 13584_2025_708_MOESM1_ESM.docx]

Appendix A. Supplementary quantitative analyses

| **Supporting Table A1**  **Participant characteristics (n=315)** |  |  |
| --- | --- | --- |
|  |  |  |
| **Variable** | **Category** | **N (%)** |
| Gender | Women | 287 (91.1) |
| District | South | 141 (44.9) |
|  | Central | 126 (40.1) |
|  | North | 47 (15.0) |
| Type of settlement | Urban | 217 (70.1) |
|  | Rural | 97 (30.9) |
| Academic education | BA | 184 (58.6) |
|  | MA | 115 (36.6) |
|  | PhD | 5 (1.6) |
|  | None | 10 (3.2) |
| Professional education | Nursing specialty | 178 (56.6) |
|  | Registered nurse | 117 (37.2) |
|  | Nurse practitioner | 16 (5.2) |
|  | Practical nurse | 3 (1.0) |
| Clinical setting | Hospital | 162 (51.4) |
|  | Primary care clinic | 63 (20.0) |
|  | Other | 90 (28.6) |
| Medic in the army | Yes | 37 (11.7) |
| Has previous experience in an emergency situation | Yes | 75 (23.8) |
| Has received training in emergency treatment | Yes | 182 (57.8) |
| Is part of an emergency team in their institution | Yes | 93 (29.5) |
| Did they respond on October 7, 2023, independently or following a call? | Yes | 78 (24.8) |
| Age | Mean (SD)  Minimum-maximum | 42.2 (12.3)  21.0-79.0 |
| Years of experience | Mean (SD)  Minimum-maximum | 14.5 (12.1)  1.0-50.0 |

| **Supporting Table A2**  **Comparison of nurses who responded and those who did not respond on Oct. 7, 2023** | | | | |
| --- | --- | --- | --- | --- |
|  | Responded (*n* = 78) | Did not respond (*n* = 223) | *t* | *p*-value |
| **Knowledge** | 3.90 (0.81) | 3.62 (0.82) | -2.62 | **0.009** |
| **Readiness** | 3.52 (0.95) | 3.20 (0.90) | -2.66 | **0.008** |
| Attitude | 4.36 (0.72) | 4.23 (0.74) | -1.38 | 0.167 |
| Norms | 3.90 (0.78) | 3.77 (0.77) | -1.22 | 0.222 |
| Self-efficacy | 3.88 (0.65) | 3.73 (0.63) | -1.79 | 0.074 |
| Hesitancy | 2.30 (0.87) | 2.40 (0.76) | 0.93 | 0.353 |
| Intention | 4.32 (0.67) | 4.22 (0.67) | -1.15 | 0.249 |
| **Personal resilience** | 3.95 (0.56) | 3.69 (0.59) | -3.30 | **0.001** |
| Community resilience | 3.35 (0.82) | 3.39 (0.78) | 0.35 | 0.728 |
| Altruism—charity | 3.42 (0.85) | 3.44 (0.79) | 0.15 | 0.881 |
| **Altruism—helping others** | 3.07 (0.87) | 2.86 (0.69) | -2.06 | **0.041** |

| **Supporting Table A3**  **Differences in the main study variables between nurses who were hesitant and those who were not** | | | | |
| --- | --- | --- | --- | --- |
|  | Hesitant nurses (*n* = 96) | Non-hesitant nurses (*n* = 219) | *t* | *p*-value |
| **Knowledge** | 3.27 (0.82) | 3.88 (0.75) | 6.50 | **<0.001** |
| **Readiness** | 2.88 (0.99) | 3.46 (0.83) | 5.34 | **<0.001** |
| **Attitudes** | 4.05 (0.79) | 4.35 (0.69) | 3.44 | **<0.001** |
| **Norms** | 3.60 (0.81) | 3.90 (0.74) | 3.25 | **0.001** |
| **Self-efficacy** | 3.55 (0.60) | 3.87 (0.62) | 4.25 | **<0.001** |
| **Intention** | 3.96 (0.70) | 4.37 (0.62) | 5.23 | **<0.001** |
| **Personal resilience** | 3.61 (0.60) | 3.81 (0.58) | 2.81 | **0.005** |
| Community resilience | 3.34 (0.78) | 3.40 (0.79) | 0.62 | 0.534 |
| Altruism—charity | 3.37 (0.76) | 3.46 (0.82) | 0.90 | 0.367 |
| Altruism—helping others | 2.81 (0.72) | 2.96 (0.75) | 1.58 | 0.115 |

| **Supporting Table A4**  **Comparison of nurses who received training and those who did not** | | | | |
| --- | --- | --- | --- | --- |
|  | Received training (*n* = 182) | Had not received training  (*n* = 132) | *t* | *p*-value |
| Knowledge | 3.82 (0.76) | 3.52 (0.88) | -3.33 | **<0.001** |
| Readiness | 3.43 (0.91) | 3.08 (0.90) | -3.44 | **<0.001** |
| Attitudes | 4.32 (0.69) | 4.19 (0.78) | -1.49 | 0.137 |
| Norms | 3.88 (0.74) | 3.71 (0.82) | -1.86 | 0.064 |
| Self-efficacy | 3.86 (0.61) | 3.66 (0.64) | -2.83 | **0.005** |
| Hesitancy | 2.31 (0.76) | 2.46 (0.81) | 1.72 | 0.086 |
| Intention | 4.33 (0.63) | 4.15 (0.71) | -2.39 | **0.018** |
| Personal resilience | 3.78 (0.58) | 3.71 (0.61) | -0.97 | 0.331 |
| Community resilience | 3.33 (0.83) | 3.44 (0.73) | 0.16 | 0.245 |
| Altruism—charity | 3.49 (0.80) | 3.36 (0.81) | -1.45 | 0.149 |
| Altruism—helping others | 2.98 (0.76) | 2.83 (0.71) | -1.79 | 0.074 |

Appendix B. Supplementary qualitative analyses

**Supporting Table B1**

**Characteristics of participants who took part in the qualitative phase**

| **Participant ID** | **Profession \ Posision** | **Gender** | **Age (years)** | **Seniority** | **Response on Oct., 7, 2023** | **Experience in prehospital care** | **Involvement in relevant policy making** |
| --- | --- | --- | --- | --- | --- | --- | --- |
| 1 | Nurse, Community setting | Female | 47 | 21 | Yes | Yes | No |
| 2 | Paramedic, Medical Officer | Male | 37 | 10 | Yes | Yes | Yes |
| 3 | Nurse, manager, Home care | Female | 56 | 30 | Yes | Yes | No |
| 4 | Nurse, Public hospital | Female | 51 | 27 | Yes | Yes | No |
| 5 | Nurse, Clinical instructor, Nursing school | Female | 42 | 17 | Yes | No | No |
| 6 | Paramedic & Researcher (Emergency medicine) | Male | 50 | 10 | Yes | Yes | Yes |
| 7 | Researcher (Emergency Preparedness and response) | Female | 44 | 8 | No | No | Yes |
| 8 | Physician & Researcher (Public health), Head School of Public Health | Female | 68 | 30 | No | Yes | Yes |
| 9 | Nurse, Coordinator for emergency preparedness | Female | 50 | 24 | No | No | Yes |
| 10 | Nurse, human resource allocation during emergencies, Ministry of Health | Female | 49 | 26 | No | No | Yes |
| 11 | Nurse, Deputy CEO of a major hospital | Female | 63 | 35 | Yes | No | No |
| 12 | Nurse, Chair, school of Nursing | Female | 63 | 42 | No | Yes | No |
| 13 | Physician, Director, Emergency room | Female | 57 | 30 | No | Yes | Yes |
| 14 | Nurse, Community setting | Female | 30 | 2 | Yes | Yes | No |
| 15 | Nurse, Community setting | Female | 63 | 35 | No | Yes | No |
| 16 | Nurse, Chair, School of Nursing | Male | 53 | 32 | No | Yes | No |
| 17 | Paramedic, Emergency Operations Manager at MDA (Israeli EMS) | Male | 36 | 8 | Yes | Yes | No |
| 18 | Physician & Researcher (Emergency medicine), Responsible for emergency preparedness at a Level I Trauma Center | Male | 65 | 33 | No | Yes | Yes |
| 19 | Paramedic, Vast experience in managing prehospital emergencies | Male | 53 | 20 | No | Yes | Yes |
| 20 | Nurse, Community setting & Clinical instructor, Nursing school | Female | 45 | 18 | Yes | No | No |
